# Newborns with Bloody Stools—At the Crossroad between Efficient Management of Necrotizing Enterocolitis and Antibiotic Stewardship

**DOI:** 10.3390/antibiotics10121467

**Published:** 2021-11-29

**Authors:** Marie Heyne-Pietschmann, Dirk Lehnick, Johannes Spalinger, Franziska Righini-Grunder, Michael Buettcher, Markus Lehner, Martin Stocker

**Affiliations:** 1 Department of Pediatric Surgery, Children’s Hospital Lucerne, 6000 Lucerne, Switzerland; markus.lehner@luks.ch; 2 Biostatistics and Methodology, Clinical Trial Unit Central Switzerland, University of Lucerne, 6000 Lucerne, Switzerland; dirk.lehnick@unilu.ch; 3 Department of Health Sciences and Medicine, University of Lucerne, 6000 Lucerne, Switzerland; 4 Division of Gastroenterology, Hepatology and Nutrition, Department of Pediatrics, Children’s Hospital Lucerne, Spitalstrasse, 6000 Lucerne, Switzerland; johannes.spalinger@luks.ch (J.S.); franziska.righini@luks.ch (F.R.-G.); 5 Division of Infectious Diseases, Department of Pediatrics, Children’s Hospital Lucerne, Spitalstrasse, 6000 Lucerne, Switzerland; michael.buettcher@luks.ch; 6 Neonatal and Pediatric Intensive Care Unit, Department of Pediatrics, Children’s Hospital Lucerne, Spitalstrasse, 6000 Lucerne, Switzerland; martin.stocker@luks.ch

**Keywords:** neonates, antibiotic stewardship, necrotizing enterocolitis, food protein-induced allergic proctocolitis, antibiotic exposure

## Abstract

The onset of bloody stools in neonates often results in antibiotic treatment for suspected necrotizing enterocolitis (NEC). Food protein-induced allergic proctocolitis (FPIAP) is an often-neglected differential diagnosis. We performed a retrospective analysis of antibiotic exposure at our tertiary center from 2011 to 2020 that included three time periods of differing antimicrobial stewardship goals. We compared these data with the conventional treatment guidelines (modified Bell’s criteria). In our cohort of 102 neonates with bloody stools, the length of antibiotic exposure was significantly reduced from a median of 4 to 2 days. The proportion of treated neonates decreased from 100% to 55% without an increase in negative outcomes. There were 434 antibiotic days. Following a management strategy according to modified Bell’s criteria would have led to at least 780 antibiotic days. The delayed initiation of antibiotic treatment was observed in 7 of 102 cases (6.9%). No proven NEC case was missed. Mortality was 3.9%. In conclusion, with FPIAP as a differential diagnosis of NEC, an observational management strategy in neonates with bloody stools that present in a good clinical condition seems to be justified. This may lead to a significant reduction of antibiotic exposure. Further prospective, randomized trials are needed to prove the safety of this observational approach.

## 1. Introduction

In neonatology units, the onset of bloody stools often results in the initiation of diagnostic procedures and preventive treatment for possible necrotizing enterocolitis (NEC). Early detection of high-risk neonates presenting with NEC as well as immediate initiation of therapy is crucial for the improvement of patients’ outcomes and mortality rates [1,2,3]. The differential diagnosis of hematochezia is broad, including life-threatening diseases such as NEC or Hirschsprung’s disease/Hirschsprung-associated enterocolitis (HAEC) or intestinal malrotation with midgut volvulus. Further, but less serious, differentials include food protein-induced allergic proctocolitis (FPIAP), food protein-induced enterocolitis syndrome (FPIES), gastrointestinal infections, anal fissures and swallowed maternal blood [1,4,5,6,7,8]. Of these, NEC and FPIAP are the most common and may serve as examples on each side of the severity spectrum.

NEC is one of the leading causes of morbidity and mortality in preterm neonates and particularly affects low birth weight (LBW) infants in neonatal intensive care units (NICUs) [1]. Its incidence in very low birth weight (VLBW) infants is estimated to be approximately 6–7%, increasing in inverse proportion to birthweight (BW) and gestational age (GA) [1,9,10]. With advances in obstetric and neonatal care, the survival rates of immature infants have improved, resulting in a population at increased risk for developing NEC [2,11,12,13,14]. Prematurity, LBW and small for gestational age (SGA) are the most consistently reported risk factors for NEC [15]. In 1987, Bell et al. proposed the first staging system for neonates with suspected NEC, which included a set of clinical manifestations to stratify patients into three different stages [16]. Almost a decade later, Kliegman et al. published a modified version of Bell’s staging criteria, including six instead of three stages, aimed at guiding therapeutic decisions based on differences of illness severity across the stages [17]. Currently, the modified Bell’s classification remains the most important and widely used staging tool for neonates with suspected NEC [18,19].

By contrast, FPIAP is a benign disease, that is mostly caused by cow milk protein or soy protein-based infant feeds [7,20,21]. Exposure and sensitization to food protein lead to gastrointestinal inflammation in the first months of life [22,23]. A typical case of FPIAP is a well-appearing breast- or formula-fed young infant with occult or even gross bloody stools. Eliminating the offending food usually results in the resolution of symptoms, proving the diagnosis [7,20,23,24].

It is challenging in a neonate with rectal bleeding to differentiate between the first symptoms of NEC and FPIAP, especially in preterm infants, where untreated NEC may lead to rapid deterioration with sepsis and the development of severe NEC. Facing this potential risk, most physicians tend to start full NEC treatment, including the initiation of antibiotic therapy and parenteral nutrition (PN) [25,26,27,28,29]. Recent studies demonstrate that the administration of antibiotics early in life leads to perturbation of the developing microbiome with potential consequences for future health [30]. In addition, every day of treatment with antibiotics itself increases the risk for NEC [31]. The aim of our study was to investigate the clinical courses of newborns with bloody stools and to analyse which patients would benefit from an early start of antibiotics and for which patients a more restrictive treatment strategy would be justified. Antibiotic exposure within three time periods of differing antibiotic stewardship goals [32] and comparison with the therapeutic guidance by the modified Bell’s staging criteria served as the primary outcome variables.

## 2. Results

A total of 114 neonates with bloody stools were hospitalized in our institution from January 2011 to June 2020. We excluded 12 neonates with anal fissures, swallowed maternal blood, gastrointestinal infection (*n* = 5), motility disorders such as Hirschsprung’s disease (*n* = 6) or isolated gastric perforation (*n* = 1). A total of 43 of the remaining 102 neonates were stratified into Bell IB (42.2%), 50 (49.0%) into Bell II (Bell IIA: 25 (24.5%), Bell IIB: 25 (24.5%)) and 9 (8.8%) into Bell III based on clinical signs as well as radiological and laboratory findings (Table 1 and Table 2).

### 2.1. Antibiotic Exposure

**Figure 1:**
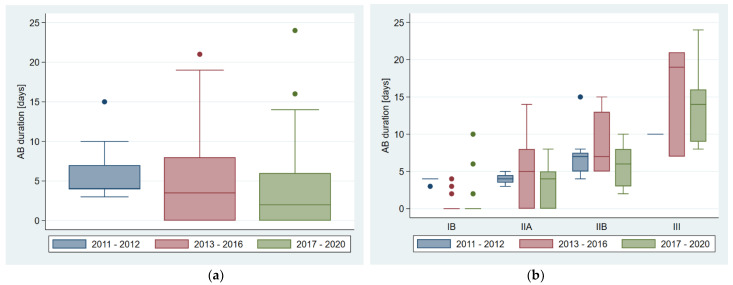
Duration of antibiotic therapy divided by time periods: (**a**) for all patients and (**b**) divided by NEC stages.

The median antibiotic exposure of all the neonates (*n* = 102) during the whole study period (2011–2020) was 4 days (range 0–24 days) (Table 2). A total of 37 neonates (36.3%) did not receive any antibiotic therapy, whereas 65 neonates (63.7%) received antibiotic therapy for a median of 6 days (range 2–24 days) (Table 2). The median antibiotic exposure of the whole cohort decreased within the study period from 4 days (2011–2012) to 2 days (2017–2020) (Figure 1a). Furthermore, the proportion of patients receiving antibiotic therapy after the onset of bloody stools declined from 100% (2011–2012) to 55.1% (2017–2020) (Table 2). Comparing the antibiotic exposure of different NEC stages within the three time periods, the duration of antibiotic treatment decreased mainly in NEC stage IB from 4 days (2011–2012) to 0 days (2017–2020). Furthermore, the proportion of neonates treated with antibiotics in this group declined from 100% (2011–2012) to 26.7% (2013–2016) to 18.2% (2017–2020), respectively (Table 2, Figure 1b). Antibiotic exposure for all neonates (*n* = 102) in our cohort summed up to 434 antibiotic days. If all the neonates had been treated according to the therapeutic guidelines of modified Bell’s staging criteria for NEC, the overall number would have resulted in at least 780 antibiotic days.

### 2.2. Outcome of Delayed Antibiotic Exposure

In seven neonates (6.9% of the whole cohort) with antibiotic exposure, treatment was not initiated immediately after symptom onset. All these patients did not show worsening of their general condition at that time. In three, antibiotic therapy was started due to the persistence of bloody stools; in the other four neonates, antibiotic therapy was started later due to radiological or laboratory findings. Antibiotic therapy was not escalated in the further course with any of these neonates and none needed surgery for NEC treatment nor died during hospitalization.

### 2.3. Mortality

Four neonates (3.9%) died during hospitalization (Table 2 and Table 3). All these infants had major comorbidities, particularly cardiac anomalies. One neonate underwent surgery for NEC after free intra-abdominal air was detected on X-ray. The other three neonates showed only minor or no radiological findings suggestive for NEC but showed general deterioration and hemodynamic instability. In all four patients, antibiotic therapy for NEC was immediately initiated after symptom onset (Table 3).

### 2.4. Feeding Regimes

The data on the feeding regimes before symptom onset were available from 95 of 102 neonates. A total of 69 out of 95 neonates (72.6%) were exclusively breast-fed, whereas 26 (27.4%) received at least partial formula-based feeds. The percentage of neonates with formula-based feeds divided by NEC stages are shown in Table 4. A change of feeds to intensive hydrolysed formula milk due to bloody stools was established in 53 of 99 neonates (53.5%) for whom data on change of feeds was captured—during the whole study period (2011–2020). From 2011–2012, only one neonate (5.3%) was changed to intensive hydrolysed formula milk, whereas from 2013–2016, and in the last period from 2017–2020, this proportion increased to 47.1% and 73.5%, respectively (Table 2). Overall, in 34 of 102 neonates (33.3%) only change of feeds was established, whereas in 19 neonates (18.6%), an antibiotic treatment was additionally started. A total of 46 patients (45.1%) only received antibiotic therapy without change of feeds and in 3 patients (2.9%), spontaneous symptom resolution occurred without any therapy.

### 2.5. Analysis of Risk Factors for NEC

The possible risk factors before symptom onset for the patients divided by NEC stages according to Bell is displayed in Table 4. Stepwise, multiple ordered logistic regression, using Akaike’s information criterion, created the best prediction model for higher NEC stages with gestational age (GA), small for gestational age (SGA), day of life (DOL) at symptoms’ onset and fresh frozen plasma (FFP) transfusion (Table 5). Therefore, higher NEC stages in our cohort of neonates with bloody stools were associated with lower GA, the presence of SGA, early onset of bloody stools, and FFP transfusion.

## 3. Discussion

During the 10 year study period, a significant reduction of antibiotic exposure in neonates with bloody stools that were hospitalized in our NICU or neonatal ward was observed without an increase of negative outcome.

While in the period of 2011 to 2012, all the neonates were treated according to therapeutic guidance by modified Bell’s criteria as suspected NEC with the administration of antibiotic therapy and parenteral nutrition, the proportion of patients with antibiotic exposure dropped significantly to 55% in the period of 2017 to 2020. Furthermore, the duration of antibiotic therapy for the whole cohort declined significantly from 4 days to 2 days. The highest reduction in antibiotic exposure was most noticeable in newborns with NEC stage IB. Generally, the reduction of antibiotic exposure was not associated with an increased risk of missing proven NEC cases or an increase in negative outcomes. Comparing antibiotic exposure according to the therapeutic guidelines of the modified Bell’s staging criteria for NEC, we were able to reduce the number of antibiotic days by at least 44%. This underlines the robustness of the observed effect and supports the generalization of our results [17]. Instead of antibiotic exposure, a change of feeds to extensive hydrolysed formula milk as therapy for FPIAP increased significantly from the first to the last study period. Nevertheless, the diagnosis of FPIAP is a diagnosis of exclusion supported by the resolution of bloody stools after a change of feeds to extensive hydrolysed formula milk. Mothers’ milk is the best food for neonates and a change to extensively hydrolysed milk is only justified if antibiotics and parenteral nutrition can be decreased, instead. A change in the mothers’ diet, abandoning milk protein, may be another strategy. Unfortunately, we do not possess detailed data about this strategy in our cohort.

The proportion and duration of antibiotic treatment, the amount of administration of parenteral nutrition, and the number of performed surgeries was higher with higher NEC stages according to Bell’s classification. This was expected and was in accordance with the therapeutic guidelines of modified Bell’s staging [17]. Mortality in our cohort was very low with 3.9%. A total of 5.8% of neonates needed surgery for NEC and the mortality rate of operated infants was 16.7%, respectively. Mortality and surgery rates remained low over the study period. In previous research, mortality in affected infants ranges from 15% to 30%, whereas 20% to 40% of neonates need surgery; among these cases, mortality rates are reported at up to 50% [1,2,3,10,33]. Therefore, compared to previous research, our study demonstrated a good outcome despite reducing antibiotic exposure significantly.

So far, there are not many studies published that question the necessity and threshold of antibiotic treatment in newborns with bloody stools. Maayan-Metzger et al. published a study comparing the management of isolated rectal bleeding (IRB) in newborns at two different time periods. They showed that a reduction in the length of antibiotic treatment and nil per os (NPO) did not lead to the deterioration of their patients’ clinical condition [34]. Lenfestey et al. published a case series of neonates that were initially diagnosed as NEC but during the further clinical course the diagnosis of food protein induced enterocolitis syndrome (FPIES) was even more likely. They commented that neonates on NICUs are often overtreated and that FPIES remains an important differential diagnosis to NEC [25]. Another case series was published by Aktas et al. They underlined that FPIAP is an important differential diagnosis in neonates with bloody stools and therefore needs to be considered more often [27].

Our study results and the observed courses of neonates with bloody stools in our cohort underline that symptoms’ onset might often not be associated with NEC but with an atypical/early-onset type of FPIAP. We therefore hypothesize an overall overtreatment of neonates with bloody stools and advocate a more restricted use of antibiotics in term and preterm newborns with bloody stools on NICUs and neonatal wards, respectively. FPIAP should be considered as primary diagnosis in neonates with bloody stools who appear to be in good clinical condition and show only minor or no abnormalities in laboratory or radiological examinations (Bell IB). Currently, antibiotic stewardship is mandatory for every neonatal unit. Nevertheless, research shows that adherence to antibiotic stewardship programs is not easy to obtain and that the fear of sepsis is a major driver for the overuse of antibiotics [35,36]. Potential sequelae of the early-life administration of broad-spectrum antibiotics are gaining importance. Facing the rising numbers of multi-drug-resistant bacteria worldwide it becomes increasingly necessary to emphasize more antibiotic use. It is postulated that antibiotics change the microbial composition of the gut as well as influencing the immune system, which may contribute to autoimmune diseases, including allergies, asthma or inflammatory bowel disease (IBD). In addition, exposure to antibiotics is also supposed to increase the risk for NEC itself [30,31,37,38,39,40,41,42]. Furthermore, the necessity to insert a central venous catheter for parenteral nutrition comes with a risk of complications, such as bloodstream infections, thrombosis, catheter dislocation/dysfunction and placement-related issues such as pneumothorax, arterial puncture, and cardiac arrhythmias [43,44].

Our secondary objective in our cohort of neonates with bloody stools was to analyse the association of risk factors with higher NEC stages. Many risk factors for NEC are correlated among each other and a bivariate analysis is problematic. Therefore, the risk factors in Table 4 may serve as a description of the population and of subgroups as NEC stages, but analysis must remain purely exploratory and hypothesis-generating. Therefore, we analysed risk factors with a multiple, ordered regression analysis, using Akaike’s information criterion to assess the correlation with higher NEC stages. Nevertheless, the prediction performance of our model was relatively modest. Low GA, SGA, early onset of symptoms (bloody stools) and FFP transfusion correlated best with higher NEC grading. Prematurity and low birth weight as well as small for gestational age (SGA) remain the most consistently proven risk factors for developing NEC in published research [15]. Therefore, it remains indispensable to consider those risk factors when assessing newborns with bloody stools. Whereas day of life at symptom onset and transfusions are also often reported among many other risk factors in previous studies, their significance remains unclear [15,45,46,47,48].

Due to the retrospective design as the main limitation of our study, we are not able to prove a causal relationship between antibiotic stewardship policies and reduced exposure to antibiotics. Nevertheless, the effect size is highly noticeable without other explanation of the observed effect. The retrospective design is even more of a limitation regarding the analysis of the risk factors of proven NEC cases. The observation of historically established risk factors and pathological diagnostics for NEC, as well as the use of Bell’s classification. may lead physicians to diagnose NEC cases and to start antibiotic treatment. Although stage III NEC may be taken as proof of NEC, there is no clear gold standard for the diagnosis. Due to safety aspects, the early administration of antibiotics for NEC stage IIA and B cases seems to be reasonable; we may challenge the indication for neonates with NEC stage IA and B. Nevertheless, due to the retrospective design, future studies are needed to prove the safety of our approach.

## 4. Materials and Methods

This is a retrospective, single-centre cohort study analysing risk factors, clinical courses, therapies, and outcome with focus on the antibiotic stewardship of neonates with bloody stools hospitalised at the Children’s Hospital in Lucerne, Switzerland. The study was approved by the national Swiss Ethics Committee, which consented to the collection of individual data.

### 4.1. Inclusion/Exclusion Criteria and Patient Stratification

Included were all newborns (gestational age < 44 0/7 weeks) with bloody stools that were hospitalized in the NICU or neonatal ward at the Children’s Hospital of Lucerne between January 1, 2011 and June 30, 2020. Neonates with apparent explanations for their bloody stools, such as anal fissures or swallowed maternal blood, as well as neonates with congenital anomalies or enteral infections, were excluded.

We classified the neonates into NEC stages I-III, according to the modified Bell’s classification of NEC [17] based on clinical signs as well as radiological and laboratory findings (Table 1). As bloody stool was set as a mandatory symptom, we did not stratify any neonates into Bell IA but at least into IB. Stages IIIA and IIIB were summarized into stage III as definitive NEC.

### 4.2. Setting and Three Different Time Periods of Antibiotic Stewardship

The Children’s Hospital of Lucerne is a tertiary neonatal centre in Central Switzerland, where birth rates are around 7000 deliveries annually. The Children’s Hospital of Lucerne is responsible for special care for all neonates, including intermediate and intensive neonatal care as well as paediatric surgery within Central Switzerland.

During the observed time period of 10 years, the management of neonates with bloody stools changed. During 2011–2012, neonates with bloody stools were always managed for a working diagnosis of NEC and classical NEC management was started until NEC was excluded. In the following years (2013–2016), there was growing evidence that NEC might not be the cause of the onset of bloody stools, but it was suggestive of an early-onset/atypical form of FPIAP and, therefore, the management changed slowly. Neonates without risk factors or clinical signs of infection, or with normal further work-up, were increasingly observed. During this period of time, antibiotic stewardship was implemented for suspected neonatal early-onset sepsis, catheter-related blood stream infections and ventilation-associated pneumonia at our centre [32]. During the period from 2017 to 2020, antibiotic stewardship was adjusted to further focus on the duration of antibiotic therapy. This was also implemented for suspected NEC. The antibiotic treatment regimen changed over the study period, aiming to reduce the use of broad-spectrum antibiotics. During 2011–2015 neonates with suspected NEC received either Amoxicillin and Meropenem or Meropenem and Vancomycin. Since 2016, we have used Amoxicillin/Clavulanic acid and Amikacin as first-line drugs and Metronidazole is added for neonates with intestinal perforation.

### 4.3. Outcomes and Objectives

The primary outcome was defined as the exposure to antibiotic treatment within three different time periods of antibiotic stewardship (2011 to 2012 versus 2013 to 2016 versus 2017 to 2020) for the whole cohort and for different NEC stages and in comparison with therapeutic guidance by the modified Bell’s staging criteria. Mortality within the three different time periods was defined as a secondary outcome.

As a secondary objective, we investigated the possible differences in risk factors between the different NEC stages according to Bell.

### 4.4. Data Acquisition and Study Variables

Neonates with bloody stools were identified through the hospital-based electronic database for diagnoses. Allergic and food-induced gastroenteritis or colitis, necrotizing enterocolitis, melena, rectal bleeding, and other diseases of the alimentary tract in newborns were used as search terms. For all the identified neonates, a retrospective chart review of the patient’ data and clinical courses was performed. The following data were collected and pseudonymised. The newborns’ clinical/birth characteristics included: gestational age (GA), birth weight (BW), gender, risk factors for NEC/comorbidities (congenital cardiac anomalies without hemodynamic relevance (e.g., small patent foramen ovale (PFO) or small patent ductus arteriosus (PDA))/with hemodynamic relevance but no need for surgery (e.g., large PDA, large ventricular septal defect (VSD))/with hemodynamic relevance and need for surgery (e.g., large PDA, double outlet right ventricle (DORV)), perinatal asphyxia, small for gestational age (SGA), sepsis, RBC (red blood cell) transfusion, PT (platelet transfusion), FFP (fresh frozen plasma) transfusion, hypotonia with need for vasopressors, antibiotic treatment before symptom onset, feeding regimen before onset). Furthermore, we evaluated the clinical symptoms (age at onset of bloody stools, change in general condition), as well as the radiological and laboratory findings and therapy (antibiotic regimen and duration, discontinuation of enteral feeding and initiation of parenteral nutrition (PN), change of feeds, surgery) and the neonates’ outcome.

### 4.5. Statistics

The data for the risk factors, therapies, and outcomes were evaluated in a descriptive manner by NEC stages. The categorial data were displayed as absolute and relative frequencies. The continuous variables were described using descriptive statistics including median, first and third quartile (Q1; Q3) and/or range (minimum–maximum). The comparisons between the risk factors and NEC stages were performed utilizing the Kruskal—Wallis test and Fisher’s exact test. Rank correlation coefficients (Kendall’s tau-b, correcting for tied ranks) were calculated to explore potential associations with increasing NEC stages. Multiple, ordered regression analysis was performed to assess potential associations of risk factors and higher NEC stages, applying Akaike’s model selection criterion. These analyses are purely exploratory and hypothesis-generating. Therefore, no adjustment for multiple testing was made. A level of significance of 0.05 was adopted. The statistical analyses were performed using Stata (Version 16.1 or later, StataCorp, College Station, TX, USA).

## 5. Conclusions

In conclusion, our study questions the necessity of antibiotic treatment for all neonates with bloody stools according to the management guidance in the modified Bell’s criteria for suspected NEC. With FPIAP as the main differential diagnosis, it seems to be justified to follow an observational management strategy in patients that appear to be in good clinical condition, which may lead to a significant reduction in antibiotic exposure. Further prospective, randomized trials are needed to prove the safety of this observational approach.

## Figures and Tables

**Table 1:** Diagnostics of patients by NEC stages.

	All Patients (*n* = 102)	Bell IB (*n* = 43)	Bell IIA (*n* = 25)	Bell IIB (*n* = 25)	Bell III (*n* = 9)
Risk Factors					
Worsening of general condition (*n*, %)	33 (32.4%)	1 (2.3%)	6 (24.0%)	18 (72.0%)	8 (88.9%)
Abdominal X-ray (*n* = 80) (*n*, %)					
—inconspicuous or minor findings	38 (47.5%)	21 (100%)	5 (20.0%)	12 (48.0%)	0 (0.0%)
— PI	35 (43.8%)	0 (0.0%)	20 (80.0%)	13 (52.0%)	2 (22.2%)
— PVG or PP	7 (8.8%)	0 (0.0%)	0 (0.0%)	0 (0.0%)	7 (77.8%)
Abdominal ultrasound (*n* = 30) (*n*, %)					
— inconspicuous or minor findings	14 (46.7%)	7 (100%)	5 (71.4%)	2 (16.7%)	0 (0.0%)
— PI	12 (40%)	0 (0.0%)	2 (28.6%)	10 (83.3%)	0 (0.0%)
— PVG or PP	4 (13.3%)	0 (0.0%)	0 (0.0%)	0 (0.0%)	4 (100%)
Blood count (*n* = 92) (*n*, %)					
— inconspicuous or minor abnormalities	74 (80.4%)	33 (100%)	24 (96.0%)	13 (52.0%)	4 (44.4%)
— suspicious for NEC	18 (19.6%)	0 (0.0%)	1 (4.0%)	12 (48.0%)	5 (55.6%)
CRP (*n* = 89), mg/L median (range)	<5 (<5–150)	<5 (<5–<5)	<5 (<5–56)	10 (<5–150)	17 (<5–76)

Abbreviations: CRP: C-reactive protein, PI: pneumatosis intestinalis, PP: pneumoperitoneum, PVG: portal-venous gas.

**Table 2:** Antibiotic exposure and outcome divided by time periods and NEC stages.

Time *p* = Period	Neonates *(n)*, Divided by NEC Stages *(n)*	Neonates with Antibiotic Therapy (*n*, %)	Duration of Antibiotic Therapy Days Median (Q1; Q3), Range	Parenteral Nutrition (PN) (*n*, %)	Change of Feeds (*n* = 99) (*n*, %)	Surgery for NEC (*n*, %)	NEC-Related Death (*n*, %)
2011–2020	Total: 102 IB: 43 IIA: 25 IIB: 25 III: 9	65 (63.7%) 13 (30.2%) 18 (72.0%) 25 (100%) 9 (100%)	4 (0; 7), 0–24 0 (0; 2), 0–10 4 (0; 5), 0–14 7 (5; 8), 2–15 14 (9; 19), 7–24	60 (58.8%) 10 (23.3%) 17 (68.0%) 24 (96.0%) 9 (100%)	53 (53.5%) 34 (79.1%) 13 (52.0%) 4 (17.4%) 2 (25.0%)	6 (5.9%) 0 (0.0%) 0 (0.0%) 0 (0.0%) 6 (66.7%)	4 (3.9%) 0 (0.0%) 0 (0.0%) 3 (12.0%) 1 (11.1%)
*p*-value Kendall’s tau-b / *p*-value		<0.001 ^a^ 0.58 / <0.001	N/A 0.65 / <0.001	<0.001 ^a^ 0.60 / <0.001	<0.001 ^a^ −0.46 / <0.001	<0.001 ^a^ 0.39 / <0.001	0.023 ^a^ 0.23 / 0.014
2011–2012 *	Total: 19 IB: 6 IIA: 4 IIB: 8 III: 1	19 (100%) 6 (100%) 4 (100%) 8 (100%) 1 (100%)	4 (4; 7), 3–15 4 (4; 4), 3–4 4 (3.5; 4.5), 3–5 7 (5; 7.5), 4–15 10 (10; 10), 10–10	17 (89.5%) 4 (66.7%) 4 (100%) 8 (100%) 1 (100%)	1 (5.3%) 1 (16.7%) 0 (0.0%) 0 (0.0%) 0 (0.0%)	0 (0.0%) 0 (0.0%) 0 (0.0%) 0 (0.0%) 0 (0.0%)	1 (5.3%) 0 (0.0%) 0 (0.0%) 0 (0.0%) 1 (5.3%)
2013–2016 *	Total: 34 IB: 15 IIA: 9 IIB: 7 III: 3	19 (55.9%) 3 (20%) 6 (66.7%) 7 (100%) 3 (100%)	3.5 (0; 8), 0–21 0 (0; 0), 0–4 5 (0; 8), 0–14 7 (5; 13), 5–15 19 (7; 21), 7–21	13 (38.2%) 1 (6.7%) 3 (33.3%) 6 (85.7%) 3 (100%)	16 (47.1%) 13 (86.7%) 3 (33.3%) 0 (0.0%) 0 (0.0%)	2 (5.9%) 0 (0.0%) 0 (0.0%) 0 (0.0%) 2 (66.7%)	0 (0.0%) 0 (0.0%) 0 (0.0%) 0 (0.0%) 0 (0.0%)
2017–2020 *	Total: 49 IB: 22 IIA: 12 IIB: 10 III: 5	27 (55.1%) 4 (18.2%) 8 (66.7%) 10 (100%) 5 (100%)	2 (0; 6), 0–24 0 (0; 0), 0–10 4 (0; 5), 0–8 6 (3; 8), 2–10 14 (9; 16), 8–24	30 (61.2%) 5 (22.7%) 10 (83.3%) 10 (100%) 5 (100%)	36 (73.5%) 20 (90.9%) 10 (83.3%) 4 (40%) 2 (40%)	4 (8.2%) 0 (0.0%) 0 (0.0%) 0 (0.0%) 4 (80%)	3 (6.1%) 0 (0.0%) 0 (0.0%) 0 (0.0%) 3 (6.1%)

Abbreviations: N/A: not applicable. ^a^ Fisher’s exact test. * Only descriptive analysis without statistical calculations in sub-groups of NEC stages due to small sample size.

**Table 3:** Mortality.

Patient No.	GA Weeks, BW Grams, APGARs’ Score	NEC Stage	Comorbidities	DOL of Symptoms’ Onset	DOL of Start of Antibiotic Therapy for NEC	DOL of NEC Surgery	DOL of Death	Cause of Death
1	28 1/7, 430 g, 1/4/6	Bell IIB	IUGR with severe cerebral, cardiac and renal anomalies; chromosomal aberration	8	8	-	13	NEC-related
2	27 4/7, 870 g, 2/6/7	Bell III	PDA with need for surgery (DOL 9)	5	5	5, 12	13	NEC-related
3	26 1/7, 870 g, 1/1/2	Bell IIB	Early onset sepsis with multi-organ failure; PFO, PDA, severe PAH, cardiac failure	7	7	-	8	NEC-related, redirection of care
4	24 2/7, 700 g, 7/8/8	Bell IIB	PDA, severe PAH and arterial hypotension; bilateral PIE	4	4	-	6	NEC-related, redirection of care

Abbreviations: BW: birth weight, DOL: day of life, GA: gestational age, IUGR: intrauterine growth restriction, PAH: pulmonal-arterial hypertension, PDA: patent ductus arteriosus, PFO: patent foramen ovale, PIE: pulmonal-interstitial emphysema.

**Table 4:** Risk factors by NEC stages.

	All Patients (*n* = 102)	Bell IB (*n* = 43)	Bell IIA (*n* = 25)	Bell IIB (*n* = 25)	Bell III (*n* = 9)	*p*-Value	Kendall’s tau-b/ *p*-Value
Risk Factors							
Gestational age (GA), weeks median (range)	33 5/7 (23 4/7–41 5/7)	34 4/7 (24 5/7–41 5/7)	34 4/7 (26 0/7–40 5/7)	30 (23 4/7–40 6/7)	33 3/7 (27 4/7–36 2/7)	0.011 ^a^	−0.22/0.003
Birth weight (BW), grams median (range)	2135 (360–4410)	2340 (1070–4130)	2260 (870–3900)	1300 (360–4410)	1680 (720–2775)	0.009 ^a^	−0.24/0.001
Sex (male/female) (*n*, %)	55 (53.9%)/ 47 (46.1%)	23 (53.5%)/ 20 (46.5%)	11 (44.0%)/ 14 (56.0%)	17 (68.0%)/ 8 (32.0%)	4 (44.4%)/ 5 (55.6%)	0.34 ^b^	−0.04/0.68
DOL at symptoms’ onset median (range)	13 (2–78)	18 (3–68)	13 (2–74)	8 (3–78)	12 (4–62)	0.043 ^a^	−0.22/0.005
Cardiac anomalies (*n*, %)	55 (53.9%)	20 (46.5%)	9 (36%)	18 (72%)	8 (88.9%)	0.001 ^b^	0.27/0.002
— without hemodynamic relevance	34 (33.3%)	16 (37.2%)	5 (20%)	11 (44%)	2 (22.2%)		
— with hemodynamic relevance	12 (11.8%)	3 (7%)	0 (0.0%)	5 (20%)	4 (44.4%)		
— with hemodynamic relevance and need for surgery	9 (8.8%)	1 (2.3%)	4 (16%)	2 (8%)	2 (22.2%)		
Asphyxia (*n*, %)	5 (4.9%)	1 (2.3%)	2 (8.0%)	2 (8.0%)	0 (0.0%)	0.68 ^b^	0.06/0.53
Sepsis (*n*, %)	6 (5.9%)	1 (2.3%)	0 (0.0%)	3 (12.0%)	2 (22.2%)	0.031 ^b^	0.21/0.023
RBC transfusion (*n*, %)	15 (14.7%)	1 (2.3%)	3 (12.0%)	8 (32.0%)	3 (33.3%)	0.001 ^b^	0.33/ <0.001
Platelet transfusion (*n*, %)	11 (10.8%)	1 (2.3%)	0 (0.0%)	8 (32.0%)	2 (22.2%)	<0.001 ^b^	0.32/ <0.001
FFP transfusion (*n*, %)	5 (4.9%)	1 (2.3%)	0 (0.0%)	2 (8.0%)	2 (22.2%)	0.041 ^b^	0.19/0.046
Arterial hypotension with need for vasopressors (*n*, %)	25 (24.5%)	6 (14.0%)	3 (12.0%)	11 (44.0%)	5 (55.6%)	0.003 ^b^	0.30/0.001
SGA (*n*, %)	7 (6.9%)	1 (2.3%)	1 (4.0%)	4 (16.0%)	1 (11.1%)	0.11 ^b^	0.18/0.052
Formula based feeds (*n* = 95) (*n*, %)	26 (27.4%)	10 (23.3%)	10 (41.7%)	6 (27.3%)	0 (0.0%)	0.19 ^b^	0.002/0.99
Antibiotic therapy before symptoms’ onset, days median (range)	4 (0–25)	3 (0–14)	3 (0–17)	4 (0–25)	4 (0–13)	0.69 ^a^	0.06/0.44

Abbreviations: DOL: day of life, FFP: fresh frozen plasma, RBC: red blood cell, SGA: small for gestational age. ^a^ Kruskal—Wallis test, ^b^ Fisher’s exact test.

**Table 5:** Stepwise multiple ordered logistic regression, using Akaike’s information criterion, for risk factors and higher NEC stages in our cohort.

Risk Factors	Odds Ratio	95%-CI	*p*-Value
Gestational age (GA)	0.84	0.77–0.92	<0.001
DOL at symptom onset	0.97	0.94–0.99	0.014
FFP transfusion	13.40	2.12–84.52	0.006
SGA	3.65	0.87–15.37	0.078

Abbreviations: DOL: day of life, FFP: fresh frozen plasma, SGA: small for gestational age.

## Data Availability

The data presented in this study are available on request from the corresponding author.
